# Prediction of the outcome of preoperative chemotherapy in breast cancer using DNA probes that provide information on both complete and incomplete responses

**DOI:** 10.1186/1471-2105-9-149

**Published:** 2008-03-15

**Authors:** René Natowicz, Roberto Incitti, Euler Guimarães Horta, Benoît Charles, Philippe Guinot, Kai Yan, Charles Coutant, Fabrice Andre, Lajos Pusztai, Roman Rouzier

**Affiliations:** 1 University of Paris – Est. ESIEE-Paris, Computer Sciences Department. Cité Descartes BP. 99, 93162 Noisy-le-Grand, France; 2 Université Paris 12, Faculté de Médecine, Institut Mondor de Médecine Moléculaire (IFR10), Créteil, F-94000, France; 3 Federal University of Minas Gerais, Brazil, Departamento de Engenharia Eletronica, Campus da UFMG (Pampulha), Av. Antonio Carlos, 6627, Belo Horizonte, MG 31270-901, Brazil; 4 University of Texas M.D. Anderson Cancer Center, Department of Breast Medical Oncology, Unit 1354, PO Box 301439, Houston, Texas, USA; 5 AP-HP, Hôpital Tenon, Department of Gynecology, 4 rue de la Chine, F-75020 Paris, France; 6 Institut Gustave Roussy, Breast Cancer Unit, 39 rue Desmoulins, 94805 Villejuif, Cedex, France; 7 UPMC Univ Paris 06, UPRES EA 4053, F-75005, Paris, France

## Abstract

**Background:**

DNA microarray technology has emerged as a major tool for exploring cancer biology and solving clinical issues. Predicting a patient's response to chemotherapy is one such issue; successful prediction would make it possible to give patients the most appropriate chemotherapy regimen. Patient response can be classified as either a pathologic complete response (PCR) or residual disease (NoPCR), and these strongly correlate with patient outcome. Microarrays can be used as multigenic predictors of patient response, but probe selection remains problematic. In this study, each probe set was considered as an elementary predictor of the response and was ranked on its ability to predict a high number of PCR and NoPCR cases in a ratio similar to that seen in the learning set. We defined a valuation function that assigned high values to probe sets according to how different the expression of the genes was and to how closely the relative proportions of PCR and NoPCR predictions to the proportions observed in the learning set was. Multigenic predictors were designed by selecting probe sets highly ranked in their predictions and tested using several validation sets.

**Results:**

Our method defined three types of probe sets: 71% were mono-informative probe sets (59% predicted only NoPCR, and 12% predicted only PCR), 25% were bi-informative, and 4% were non-informative. Using a valuation function to rank the probe sets allowed us to select those that correctly predicted the response of a high number of patient cases in the training set and that predicted a PCR/NoPCR ratio for validation sets that was similar to that of the whole learning set. Based on DLDA and the nearest centroid method, bi-informative probes proved more successful predictors than probes selected using a t test.

**Conclusion:**

Prediction of the response to breast cancer preoperative chemotherapy was significantly improved by selecting DNA probe sets that were successful in predicting outcomes for the entire learning set, both in terms of accurately predicting a high number of cases and in correctly predicting the ratio of PCR to NoPCR cases.

## Background

The development of high-throughput measurement technologies and associated computational analysis tools allow tumors to be identified based on a profile of mRNA expression levels [1-13]. Currently, most DNA chips contain more than 20 000 probe sets. These expression profiles obtained from biopsies or fine needle aspirations can then be correlated with traditional tumor characteristics (size, grade) and behaviour (recurrence, sensitivity to treatment). In breast cancer, neoadjuvant chemotherapy, which is treatment provided prior to surgery, allows breast tumor chemosensitivity to be tested in vivo [11,14-16]. A pathologic complete response (PCR) at surgery correlates with an excellent outcome, whereas residual disease (NoPCR) is associated with a poor outcome. Investigators have reported the use of gene profiling of tumors and multigene predictors (signatures) to predict response to treatment. Accurate prediction of tumor sensitivity to preoperative chemotherapy is important because NoPCR patients could be spared ineffective treatment and instead be administered alternative treatments. Therefore, such predictors allow for the delivery of individualized treatments [9,16,17].

The design of such a multigene predictor of patient class (PCR or NoPCR) involves the use of a learning data set, in which the cases have been divided into two groups according to the known outcome of the treatment, and of an independent validation set.

Three main challenges arise when designing such predictors [10,12,18-22]:

• selecting subsets of DNA probe sets relevant to the pathology and to the preoperative chemotherapy

• combining the mRNA expression levels of these subsets of DNA probe sets in order to get a reliable prediction of the efficacy of the preoperative chemotherapy

• ensuring that the performance of the predictor is independent of the learning data set (in other words, estimating the accuracy of future predictions)

The most commonly used methods for selecting a subset of DNA probe sets identify probes that deviate most from a random distribution of expression levels or that are the most differentially expressed between the PCR and NoPCR learning cases. In the former approach, statistical analysis is used to rank genes based on the calculated p-values of the probe sets, and this ranking provides the basis for gene selection [18,20-23].

In this study, we hypothesized that multigenic predictor performance could be improved if it were based on probe sets whose individual predictions were close to those of a hypothetical ideal probe set. First we considered single probe sets and their individual predictions of treatment outcomes. Then we used a valuation function to assign high values to probe sets that correctly predicted many cases in the learning set, and that predicted relative proportions of PCR and NoPCR cases close to those of the whole learning set.

We compared the performance of multigenic predictors using the 30 probe sets showing the highest p-values in t tests and the highest results for the valuation function.

## Results

### Top-ranked probe sets

We calculated the valuation of the 22 283 probe sets contained in the microarrays and ranked the probe sets according to their *v(s) *values. Table 1 gives, for each of the 30 top ranked probe sets, the corresponding gene, the probe set valuation, the number *p(s) *of correctly predicted PCR learning cases, the number *n(s) *of correctly predicted NoPCR learning cases, and the total number *c(s) *= *p(s) *+ *n(s) *of correctly predicted learning cases.

**Table 1:** Top 30 probes. Thirty probes of highest value v(s). 1st column: gene name in Hugo Gene nomenclature; 2nd column: reference of the Affymetrix DNA probe; 3rd column, v(s): probe valuation; 4th to 6th columns, p(s), n(s), c(s): numbers of pcr and nopcr correct predictions and total number c(s) = p(s) + n(s) of correct predictions for the 21 PCR and 61 NoPCR cases of the learning set.

**Gene**	**Probe**	**v(s)**	**p(s)**	**n(s)**	**c(s)**	**p value (t test)**	**rank among probe sets according to p value by t test**
**BTG3**	213134_x_at	0.61	12	40	52	2,96E-05	58
**BTG3**	205548_s_at	0.61	12	40	52	3,31E-05	62
**GATA3**	209604_s_at	0.59	15	29	44	0,00020015	148
**GATA3**	209603_at	0.49	12	26	38	0,00029983	186
**THRAP2**	212207_at	0.46	8	34	42	2,56E-08	3
**SCCPDH**	201826_s_at	0.46	12	22	34	0,00033299	194
**SIL**	205339_at	0.45	10	27	37	0,00049604	233
**KRT7**	209016_s_at	0.45	6	38	44	0,00248585	539
**MCM5**	201755_at	0.45	7	35	42	0,00281786	567
**NME3**	204862_s_at	0.44	10	25	35	2,45E-05	52
**METRN**	219051_x_at	0.44	11	22	33	1,71E-06	19
**PDE4B**	211302_s_at	0.43	9	27	36	0,00824095	1062
**PHF15**	212660_at	0.42	7	32	39	2,83E-05	57
**SSR1**	200891_s_at	0.42	7	32	39	0,00187004	466
**PISD**	202392_s_at	0.42	11	20	31	6,61E-05	92
**MELK**	204825_at	0.41	8	28	36	0,00012238	120
**CA12**	215867_x_at	0.41	10	22	32	4,42E-05	74
**CA12**	214164_x_at	0.41	10	22	32	4,70E-05	77
**MAPK3**	212046_x_at	0.41	10	22	32	9,07E-06	31
**GATA3**	209602_s_at	0.41	13	13	26	0,00068129	269
**BBS4**	212745_s_at	0.41	3	42	45	1,25E-07	4
**DAPK1**	203139_at	0.41	9	24	33	0,0006761	266
**SAS**	203226_s_at	0.40	7	29	36	8,70E-05	106
**FLJ10916**	219044_at	0.40	8	26	34	1,30E-06	15
**E2F3**	203693_s_at	0.40	8	26	34	0,00044243	219
**AHNAK**	220016_at	0.40	9	23	32	0,0001449	129
**KLHDC3**	214383_x_at	0.40	9	23	32	0,00071442	279
**SFRS12**	212721_at	0.40	9	23	32	5,34E-05	87
**SRPK1**	202200_s_at	0.39	6	31	37	0,00067356	265
**CXCR4**	217028_at	0.39	8	25	33	0,00098699	339

For instance, each of the two probe sets of gene BTG3 correctly predicted the outcome of 12 of 21 PCR learning cases and 40 of 61 NoPCR cases.

Since the valuation function based on the mean and standard deviation of gene expression level, we used a t test to determine the p values of gene expressions for PCR and NoPCR for the 30 top-ranked probe sets. The p values ranged from 2.56 × 10^-8 ^to 0.008.

The ranks of these p-values for the 30 probe sets with the highest valuation functions among the 22 283 probe sets ranged from 3 to 1062 (median: 124). The 30 probe sets with the highest p-values shared eight probe sets in common (Table 2).

**Table 2:** Valuation of the probes selected by K. Hess & al. Top 30 probes of K. Hess & al. [1]. 3rd and 4th columns: probes' values *v(s) *and ranks in this valuation. 5th and 6th columns: numbers of pcr and nopcr predictions of the probes. Total numbers of pcr and no pcr predictions: 123 and 894. Ratio = 0.13

**Gene**	**Probe**	**v(s)**	**rank**	**p(s)**	**n(s)**
**MAPT**	203929_s_at	0.22	780	0	28
**MAPT**	203930_s_at	0.291	218	2	30
**BB_S4**	212745_s_at	0.41	21	3	42
**MAPT**	203928_x_at	0.22	781	0	28
**THRAP2**	212207_at	0.46	5	8	34
**MBTP_S1**	217542_at	0.26	391	0	32
**MAPT**	206401_s_at	0.22	900	0	27
**PDGFRA**	215304_at	0.32	118	4	28
**ZNF552**	219741_x_at	0.24	564	1	27
**RAMP1**	204916_at	0.22	774	0	28
**BECN1**	208945_s_at	0.30	165	4	26
**BTG3**	213134_x_at	0.61	1	12	40
**SCUBE2**	219197_s_at	0.15	3078	0	19
**MELK**	204825_at	0.41	16	8	28
**BTG3**	205548_s_at	0.61	2	12	40
**AMFR**	202204_s_at	0.23	662	0	29
**CTNND2**	209617_s_at	0.27	337	0	33
**GAMT**	205354_at	0.38	38	7	27
**CA12**	204509_at	0.24	566	1	27
**FGFR1OP**	214124_x_at	0.37	52	6	28
**KIAA1467**	213234_at	0.25	475	3	22
**METRN**	219051_x_at	0.44	11	11	22
**FLJ10916**	219044_at	0.40	24	8	26
**E2F3**	203693_s_at	0.40	25	8	26
**ERBB4**	214053_at	0.21	1040	0	26
**JMJD2B**	215616_s_at	0.37	45	7	26
**RRM2**	209773_s_at	0.37	51	3	37
**FLJ12650**	219438_at	0.27	293	0	34
**GFRA1**	205696_s_at	0.18	1994	0	22
**IGFBP4**	201508_at	0.38	39	7	27

**Figure 1:**
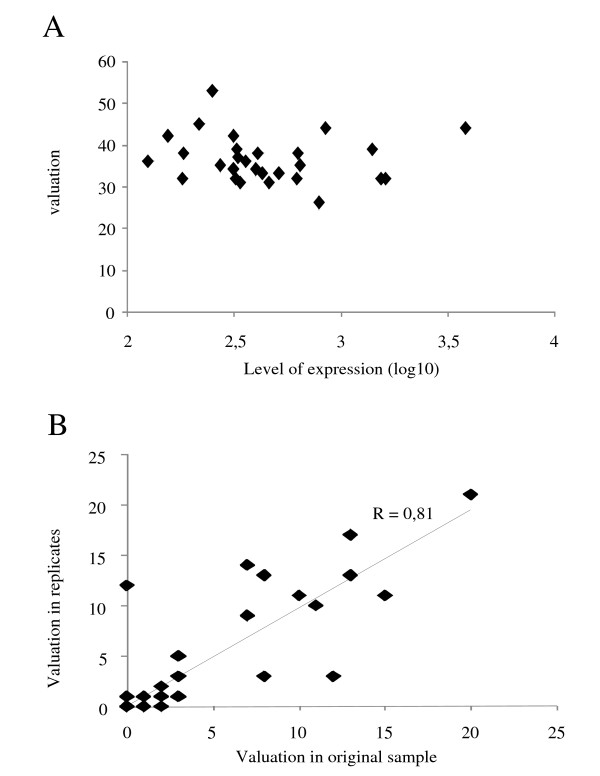
A. correlation between the level of expression of the probe sets and valuation function; B. correlation of valuation function in 30 replicates.

We then studied whether there was a correlation between the level of expression of the probe sets and their valuation. As shown in Figure 1A, we did not find any correlation (r = 0.1), suggesting that the valuation function does not depend on the level of expression of the probe set.

We also studied the correlation of valuation function in 30 replicates. In Figure 1B, we report the correlation of the valuation function for the 30 top-ranked probe set. We did not re-rank all the probe sets because the number of PCR cases (6) was relatively low in this particular subset. The correlation between original samples and replicates was high: r = 0.81. When p-values obtained by t-test were compared between replicates, the correlation was r = 0.87. This demonstrates that our method is reproducible.

#### Bi-informative and mono-informative probe sets

The definition of the pcr and nopcr predictions of a probe set leads to three different kinds of probe sets:

• the bi-informative probe sets: each of them predicts at least one PCR learning case and one NoPCR learning case, i.e. *p(s)*>0 and *n(s)*>0;

• the mono-informative probe sets: each of them is informative of a single class of patient cases:

◦ PCR-probe sets: *p(s)*>0 and *n(s) *= 0,

◦ NoPCR-probe sets: *n(s)*>0 and *p(s) *= 0;

• the non-informative probe sets: *p(s) *= *n(s) *= 0.

**Figure 2:**
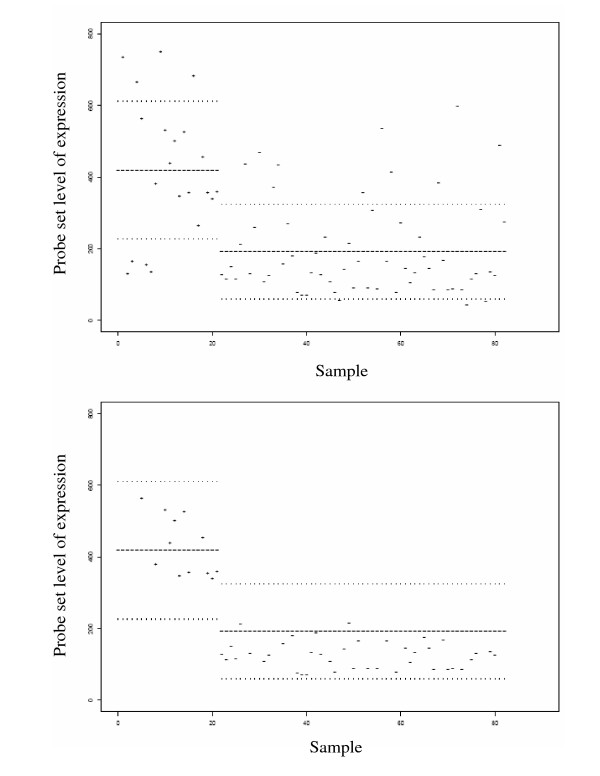
expression levels of a bi-informative probe set, probe *s *= 213134_x_at of gene BTG3, for the 82 cases of the learning set.

Figures in additional file 1 and Additional file 2 illustrate a bi-informative probe set, a PCR probe set, and a NoPCR probe set. In the upper part of Figure 2, the expression levels of a bi-informative probe set, probe *s *= 213134_x_at of gene BTG3, is shown for the 82 cases of the learning set.

The expression levels of the 21 PCR patient cases are plotted with the character "+" and those of the 61 NoPCR patient cases with the character "-". The interval of PCR expression levels *I*_*p*_*(s) *is represented by three lines of height *m*_*p*_*(s)*, *m*_*p*_*(s)*-*sd*_*p*_*(s) *and *m*_*p*_*(s)*+*sd*_*p*_*(s) *drawn based on the expression levels of the PCR learning cases.

The interval of NoPCR expression levels *I*_*n*_*(s) *is represented by lines of heights *m*_*n*_*(s)*, *m*_*n*_*(s)*-*sd*_*n*_*(s) *and *m*_*n*_*(s)*+*sd*_*n*_*(s) *drawn based on the expression levels of the NoPCR learning cases.

The lower part of Figure 2 shows the pcr and nopcr predictions of the probe set. One can see that the probe set of gene BTG3 predicted the treatment's outcome of 12 PCR learning cases and 40 NoPCR learning cases.

Using the same representation system, additional file 1 reports the PCR probe set *s *= 213033_s_at of gene NFIB, and additional file 2 reports the NoPCR probe set *s *= 203928_x_at of gene MAPT. The former predicted the treatment's outcome of 13 PCR learning cases; the latter predicted the outcome of 28 NoPCR learning cases.

For the learning data set, the proportion of the 22 283 probe sets belonging to each of the three types of probe sets were as follows:

• mono-informative probe sets: 71% (59% NoPCR probe sets and 12% PCR probe sets);

• bi-informative probe sets: 25%;

• non-informative probe sets: 4%.

In spite of the high proportion of mono-informative probe sets, none of them was found among the set of 30 top-ranked probe sets. In fact, the first mono-informative probe set was ranked at position 63: NoPCR probe set *s *= 207067_s_at of gene HDC.

This is the direct result of the valuation function *v(s) *for the probe set. It is not an arbitrarily imposed requirement of our analysis.

We have investigated the *informativity *of the probe sets with the highest p-values by t-test and it appeared that this property is a characteristic of our method. In the study of Hess et al. [1], which provided the data for the present work, the probe sets were ranked according to the p-value calculated from a t test. Of the 30 highest-ranking probes in that study, 11 are NoPCR-probe sets (Table 2).

#### Ratio of pcr to nopcr correct predictions

The ratio of the PCR to NoPCR cases (*P/N*) in the learning set was

*P*/*N *= 21/61 = 0.34

**Figure 3:**
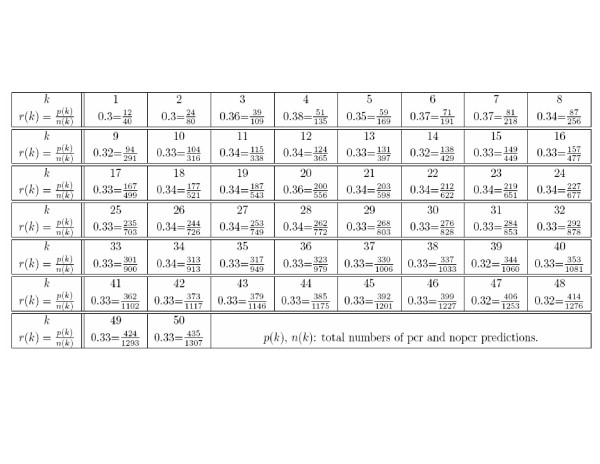
Ratios of pcr to nopcr predictions of the *k *top-ranked probes, 1 ≤ *k *≤ 50.

This ratio is in excellent agreement with the ratios of the total number of pcr and nopcr correct predictions using the *k *top-ranked probe sets; the ratios for *k *= 1 to 50 lay between 0.30 and 0.38 (Figure 3). This result confirmed that the predictions of the high-ranking probe sets were close to those of the ideal probe set not only in the number of learning cases they correctly predicted, but also in the ratio of PCR to NoPCR cases among these predicted cases.

These results also seemed to be a particular feature of our method of probe selection: in the study reported by Hess et al. [1], the ratio of pcr to nopcr predictions of the 30 probe sets with the highest p-values was 123/894 = 0.13. This ratio was three times lower than the ratio of PCR to NoPCR cases in the learning set. These ratios were, in turn, very close to that of correct pcr to nopcr predictions for all probe sets: 22 925/180 874 = 0.13.

### Multigenic predictors

#### Internal validation of k probe set predictors

We first evaluated the performance of multigenic predictors in a leave-one-out cross validation and in a k-fold cross-validation (k = 3). For the leave-one-out cross-validation, we repeated the probe selection for each procedure. For the k-fold cross-validation, we used the 30 highest-ranking probes in order to investigate whether the importance of probe selection is important for every method used to construct the multigenic predictor. We investigated DLDA, and nearest centroids. The p-values of methods in cross-validation procedures were based on 1000 random permutations.

The results indicated in Table 3 showed that DLDA had similar performance with t-test probe sets and bi-informative probe sets (mean percentage of correctly classified tumors: 82% in LOOCV, and 83% in 3-fold cross validation). Bi-informative probes improved the nearest centroid method.

**Table 3:** Cross-validation of multigenic predictors

**Leave-one out cross-validation**				
	30 t-test probes		30 bi-informative probes	
	
	Mean percent of correct classification	P value	Mean percent of correct classification	P value
**Majority vote**	-	-	73	<0.01
**DLDA**	82	<0.01	82	<0.01
**nearest centroids**	63	0.09	70	<0.01

**k-fold cross validation, k = 3**				
	30 t-test probes		30 bi-informative probes	
	
	Mean percent of correct classification	P value	Mean percent of correct classification	P value

**Majority vote**	-	-	65	<0.01
**DLDA**	83	<0.01	83	<0.01
**nearest centroids**	65	0.09	70	<0.01

#### External validation: independent datasets

**Figure 4:**
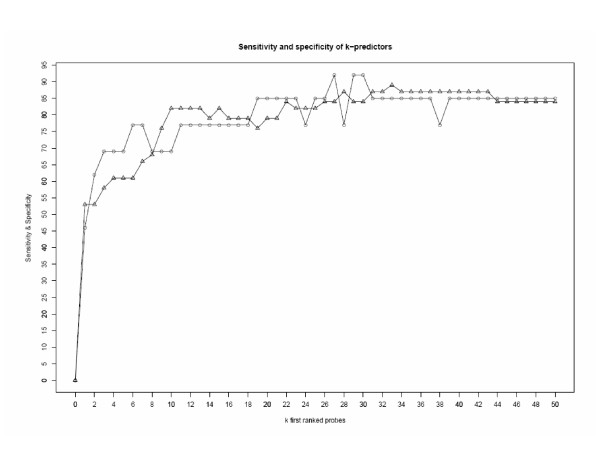
Sensitivity and specificity of the first 51 k probe set majority vote predictors (0 ≤ *k *≤ 50).

The first set of validation cases (same patient characteristics, same treatment) contained data of 51 patients, and the response to treatment was PCR for 13 patient cases and NoPCR for 38 patients. Hence, the ratios of PCR to NoPCR patient cases were the same for the learning and the validation datasets. Figure 4 depicts the values of sensitivity and specificity of the first 51 k probe set majority vote predictors (0 ≤ *k *≤ 50). The 29 and 30 probe set predictors achieved the performances of the 27 probe set predictor. Table 4 shows the numbers of false positives and false negatives for the k probe sets predictors (0 ≤ *k *≤ 50) for the external validation data. The 27 probe set predictor misclassified a total of seven patient cases: one was a false negative and six were false positives^2^. This result confirms that in this population, a 30-probe set predictor provides the most accurate results [1].

**Table 4:** Numbers of false positives and false negatives for the k probe sets predictors (0 ≤ *k *≤ 50) for the external validation data (test set 1)

**k**	**FP**	**FN**	**FP+FN**	**k**	**FP**	**FN**	**FP+FN**	**k**	**FP**	**FN**	**FP+FN**
**0**	38	13	51	17	8	3	11	34	5	2	7
**1**	18	7	25	18	8	3	11	35	5	2	7
**2**	18	5	23	19	9	2	11	36	5	2	7
**3**	16	4	20	20	8	2	10	37	5	2	7
**4**	15	4	19	21	8	2	10	38	5	3	8
**5**	15	4	19	22	6	2	8	39	5	2	7
**6**	15	3	18	23	7	2	9	40	5	2	7
**7**	13	3	16	24	7	3	10	41	5	2	7
**8**	12	4	16	25	7	2	9	42	5	2	7
**9**	9	4	13	26	6	2	8	43	5	2	7
**10**	7	4	11	27	6	1	7	44	6	2	8
**11**	7	3	10	28	5	3	8	45	6	2	8
**12**	7	3	10	29	6	1	7	46	6	2	8
**13**	7	3	10	30	6	1	7	47	6	2	8
**14**	8	3	11	31	5	2	7	48	6	2	8
**15**	7	3	10	32	5	2	8	49	6	2	8
**16**	8	3	11	33	4	2	6	50	6	2	8

In the present article, we have decided to use a very simple classification criterion for defining the k probes predictors, namely, unweighted majority voting among the predictions of the probes. Many other classifiers could be developed for the selected probes, and countless studies have been devoted to this issue. Hess et al. [1] studied several of them using a varying numbers of probes and a total of 780 classifiers (sets of genes and classifying methods). These classifiers were composed of probes selected according to their p-value calculated from a t-test. The researchers showed that among these predictors, the one showing the best performance for these particular data was the diagonal linear discriminant analysis with 30 probe sets (DLDA-30 predictor).

**Figure 5:**
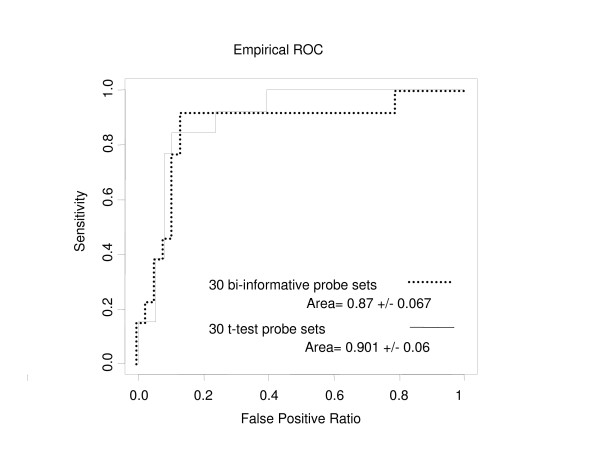
Discriminations of the two DLDA classifiers (30 probes with the highest valuation functions, and 30 probe sets showing the highest p-values (t-test)) in the independent test set 1.

**Figure 6:**
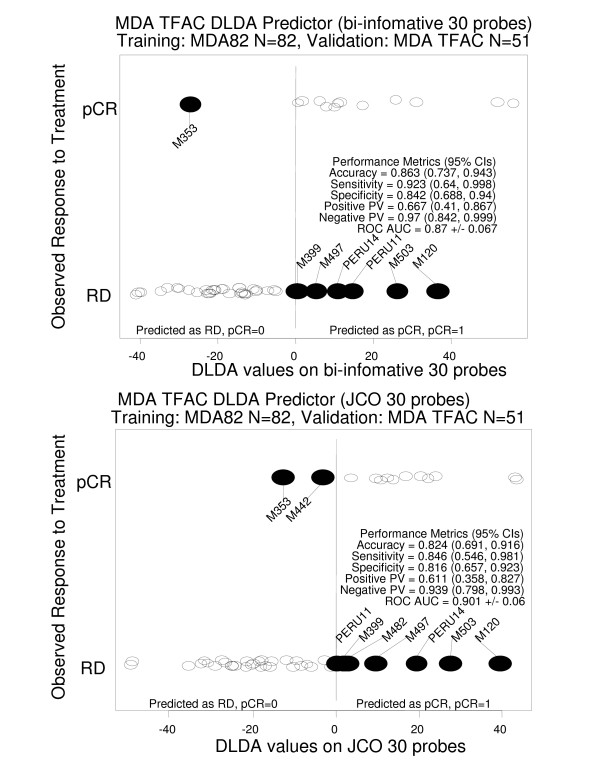
Performance metrics of the two DLDA classifiers (30 probes with the highest valuation functions, and 30 probe sets showing the highest p-values (t-test)) in the independent test set 1.

We have evaluated the DLDA classifier composed of the 30 probes with the highest valuation functions, and compared it to a DLDA classifier composed of the 30 probe sets showing the highest p-values. The discriminations of the two classifiers are represented in Figure 5: AUC obtained with the bi-informative probes and the t-test probes were 0.87 +/- 0.07 and 0.90 +/- 0.06, respectively. The performance metrics analysis (Figure 6) showed that the DLDA classifier built from the bi-informative probes had a better accuracy (0.863, 95% confidence interval: 0.737–0.943) than the t-test DLDA-classifier (0.824, 95% confidence interval: 0.691, 0.916). Interestingly, misclassified cases were similar between the two probe sets. The DLDA predictor built from the bi-informative probes correctly classified two additional patients compared to the DLDA predictor built with the t-test probes. The DLDA classifier built with the bi-informative probes had a better sensitivity (0.923, 95% confidence interval: 0.64–0.998) than the t-test DLDA-classifier (0.846, 95% confidence interval: 0.546, 0.981).

**Figure 7:**
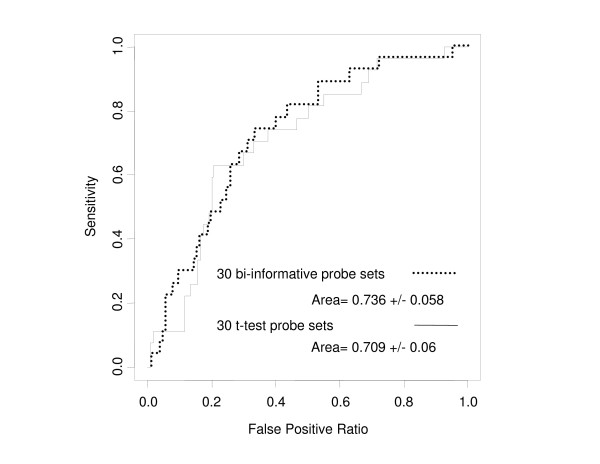
Discriminations of the two DLDA classifiers (30 probes with the highest valuation functions, and 30 probe sets showing the highest p-values (t-test)) in the independent test set 2.

**Figure 8:**
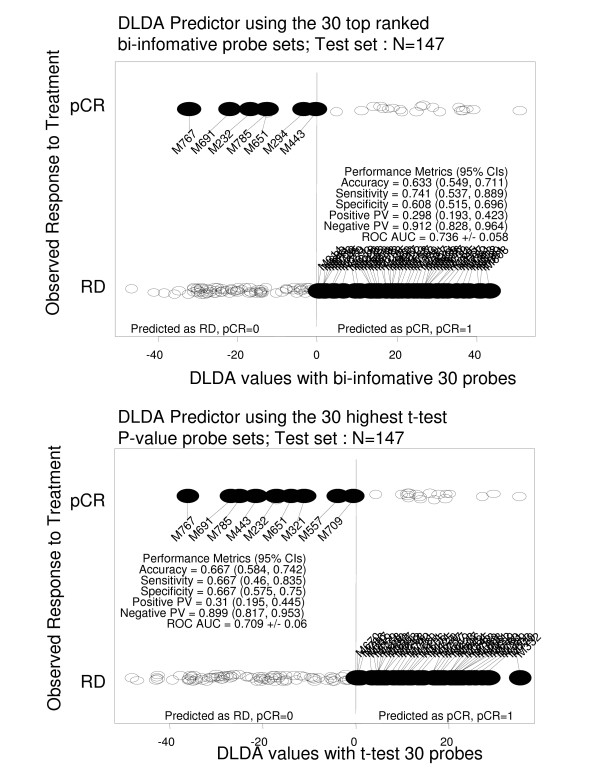
Performance metrics of the two DLDA classifiers (30 probes with the highest valuation functions, and 30 probe sets showing the highest p-values (t-test)) in the independent test set 2.

The second set of validation consisted of 147 patients treated with the same chemotherapy regimen as the learning set, but very few of these patients had a tumor with HER2 amplification. The discriminations of the two classifiers are represented in Figure 7: AUC obtained with the bi-informative probes and the t-test probes were 0.736 +/- 0.058 and 0.709 +/- 0.06, respectively. The performance metrics analysis (Figure 8) showed that the DLDA classifier built with the bi-informative probes was slightly more sensitive (0.741, 95% confidence interval: 0.537, 0.889) than the t-test DLDA-classifier (0.667, 95% confidence interval: 0.46, 0.835). This suggests that the "positive" informativity of bi-informative probes may translate into greater sensitivity.

The third validation set consisted of 50 patients treated with anthracycline-based neoadjuvant chemotherapy. Discriminations were poorer compared to previous validation sets (see Additional file 3): AUC obtained with the bi-informative probes and the t-test probes were 0.654 +/- 0.078 and 0.643 +/- 0.079, respectively. The performance metrics analysis showed that the DLDA classifier built with the bi-informative probes were more accurate (0.54, 95% confidence interval: 0.39, 0.68) than the t-test DLDA-classifier (0.52, 95% confidence interval: 0.37, 0.68). The sensitivity was the same for both probe set selection methods (0.875, 95% confidence interval: 0.6764, 0.9734). This combination of low accuracy and high sensitivity suggests that multigenic predictors are at least partly specific of a chemotherapy regimen, and that they are sensitive to the ratio of PCR and NoPCR.

#### P-value of the majority vote predictors

The p-values of the 27, 29, and 30 probe set predictors were less than 1.12 × 10^-12^, based on the null hypothesis of a predictor composed of random probe sets. Individual probe set predictions were chosen at random among the three possible values, pcr, nopcr, and unspecified, with probabilities coming from the validation set data. The details of the computation of the upper bound of the p-values are in the Appendix "P-value of the predictors" [see Additional file 4].

#### Weighting the predictions of probes

We defined a family of valuation functions, *v*_*α*_*(s)*, parameterized by the real number alpha, *α *∈ [0, 1]:

$${\text{v}}_{\alpha }\text{(s) }=\alpha \times \left(\frac{p(s)}{P}\right)+(1-\alpha )\times \left(\frac{n(s)}{N}\right)$$

**Figure 9:**
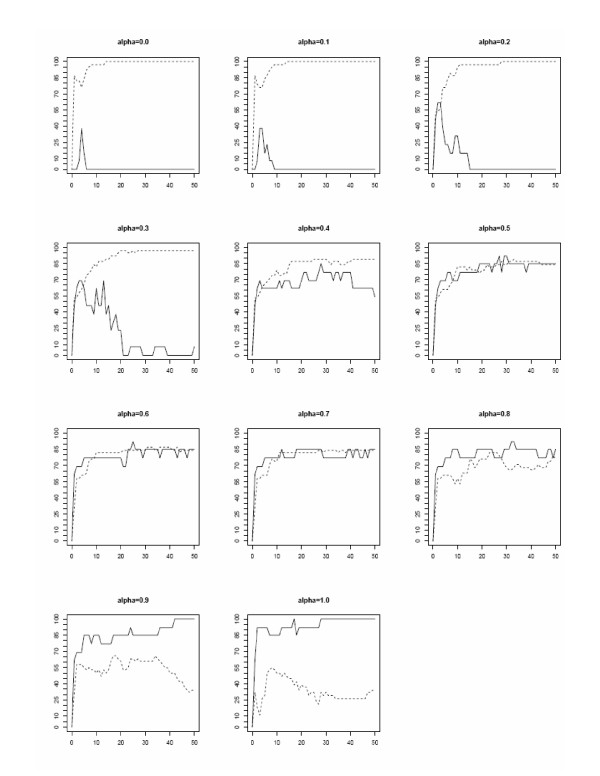
**Sensitivity and specificity of the predictors with weighted valuation function *v*_*α*_*(s)*.** Sensitivity and specificity of the k-probes predictors, 0 ≤ *k *≤ 50, for weightings *α*, *α *∈ {0, 0.1,...,1.0}. Sensitivity: continuous lines, specificity: broken lines.

The valuation function *v(s) *previously defined is the particular case *v*_*α*_*(s)=*0.5*(s)*. High parameter values favor probes with high numbers of pcr predictions *p(s) *and *vice-versa*. The valuation of a probe depends on the parameter *α*, so its rank depends on this parameter as well as on the set of *k *top-ranked probes, hence the *k *probe predictors. For each value *α *∈ {0, 0.1,...,1.0}, additional file 5 gives the set of 30 top-ranked probes and the performances of the predictor composed of the 30 top-ranked probes based on the valuation function *v*_*α*_*(s)*. Additional file 6 gives the ratios of pcr to nopcr predictions for this weighted valuation functions. Additional file 7 provides sets top 30 probes for the weighted valuation functions. Figure 9 depicts how the sensitivity and specificity of the 50 first *k *probe predictors varied with the values of the weighting parameter *α *∈ {0, 0.1,...,1.0}. The parameter value *α *= 1 grants all the weight to the pcr predictions *p(s) *of the probes. For *α *= 1, all *k *probe predictors with *k *≥ 15 classified any patient case as PCR (sensitivity = 1, specificity = 0). In contrast, the parameter value *α *= 0 grants all the weight to the nopcr predictions *n(s)*. The specificity of the resulting predictors was 1 (all the NoPCR cases were correctly predicted) and their sensitivity was very low (almost all the PCR patient cases were misclassified). For this set of 10 parameter values, the parameter value *α *= 0.5, which is given by the initial valuation function *v(s)*, provides the best 30 probe predictor. Only one other value of *α *(0.4), yielded a 30 probe predictor with the same accuracy value of 0.86, but it had a lower sensitivity.

The explanation of these results lies in the ratio *R*(*α*) of pcr to nopcr numbers of predictions for the top 30 probes in the ranking of the function *v*_*α*_*(s)*. The values of these ratios *R*(*α*) should be compared to the ratio *R *= *P/N *= 0.34 of the numbers of PCR to NoPCR learning cases. Additional file 6 gives these ratios *R*(*α*) for *α *∈ {0, 0.1,...,1.0}. The set *S*_*α *_of the top 30 probes for the weighting parameter *α *= 0.5 had a ratio *R*(*α*) = *P*(*α*)/N(*α*) = 0.33, where *P*(*α*) and *N*(*α*) are the total numbers of pcr and nopcr predictions of the set of probes *S*_*α*_. This value was the closest to the ratio *R *= 0.34. The ratio *R*(*α*) increases with the parameter *α*, from R(0) = 0.036 to *R*(1) = 1.015, which are far lower and far higher, respectively, than the ratio *R *= 0.34. These values explain the performances of the respective predictors. The conclusion is that the valuation function *v(s) *= *v*_0.5_*(s) *gives the best predictor. Nevertheless, the weighting can be used to favor specificity or sensitivity of the predictors.

## Discussion

We have introduced a new procedure to select probes that can be used as multigenic predictors. This procedure selects probes that convey information on both positive and negative issues. Using cross-validation, we have confirmed that predictors built with bi-informative probes provide similar results as predictors built with probes selected using a t test. Predictors with bi-informative probes perform better on independent datasets.

One crucial problem in predicting cancer prognosis based on microarray data is that of building prediction models based on ~50 genes that are stable in both the learning set and the actual sample set. The most common approach is to consider each probe set individually and see whether it distinguishes samples with different class labels by using Student's t test (univariate parametric significance level). This is a simple method for testing whether two variables have identical Gaussian distributions. Generally, a significance level is chosen for determining the genes that will be included in the predictors; genes that are differentially expressed between the classes at less than the specified threshold are included in the predictor. Therefore, all genes are ranked according to the result and the top k genes are selected as the feature subset to be used. In our study, for example, we selected the 30 top-ranked genes because Hess et al determined that 30 probe sets were optimal [1].

Michiels et al. [24] have analyzed seven published microarray cancer data sets, and highlighted the difficulties inherent to this approach. Examining different prostate cancer data sets, Wang et al. [10] found that misclassification rates strongly depended on which samples were used for training and which probes were selected for predictor construction. There is therefore a need to find robust gene selection methods for multigenic predictors.

There are several methods to select probes that could be of interest for a multigenic predictor: these methods may be based either on biological aspects, computational aspects or, as in the present study, on the samples themselves.

Paik et al. [6], for example, have selected probes for genes previously demonstrated to be important in breast cancer in order to predict survival and response to adjuvant chemotherapy. We tried to use these genes, but obtained unsatisfactory results (data not shown). Indeed, most of these genes proved monoinformative and captured a very singular aspect of breast tumors. Because probe sets may be redundant, some authors have reported a way to remove redundancy in the selected gene set that is compatible with any method [25]. These biological aspects, while interesting when considering targeted therapy such as estrogen receptor expression for hormone therapy or HER2 amplification for trastuzumab, do not provide more information than classic biomarkers in the case of non-targeted therapy such as chemotherapy.

Other groups have reduced the dimensionality by singular value decomposition (SVD), also referred to as principal component analysis (PCA), using, for example, the first ten principal components or metagenes to build predictors [26,27]. Bo and Jonassen have developed the "greedy-pairs method" for selecting genes [28]. In this approach, all genes are ranked based on their individual t-scores on the training set. The procedure selects the highest-ranked gene g_i _and finds the one other gene g_j _that, together with g_i_, provides the best discrimination. This is measured using the distance between centroids of the two classes with regard to the two genes when projected on the diagonal linear discriminant axis. These two selected genes are then removed from the gene set and the procedure is repeated on the remaining set until a specified number of genes has been selected. This method attempts to select pairs of genes that work well together to discriminate the classes. It is computationally efficient, but it rarely reveals biological characteristics.

In order to maximize the information provided by the training set, some methods are based on results provided by internal cross-validation. Wang et al. have reported a greedy robust feature selection approach built on the leave-one-out cross-validation procedure to retain the most frequently identified genes for building a predictive model [10]. Leaving out one sample at a time, they used a greedy-LDA to identify a set of predictive genes. They counted the number of times a gene was selected, and retained only the most frequently identified genes as the selected features. Similarly, Michiels et al. proposed performing repeated random sampling to improve the stability of predictors [24]. Jiang et al. proposed a gene shaving method based on Random Forests and another method based on Fisher's Linear Discrimination, leading the researchers to discover marker genes with expression patterns that could differentiate lung cancer cells from normal cells [29]. Fisher's Linear Discrimination is a traditional classification method that is computationally efficient, while Random Forests is based on growing an ensemble of trees (classifiers) on bootstrapped samples, which significantly improves the classification accuracy.

Our approach is somehow different because it favors probes that convey information on samples with positive and negative outcomes. All probes are differentially expressed in both subsets of patients; in fact, our method assumes that samples of different classes have Gaussian distributions, as demonstrated by p values calculated by t test. The probes are subjected to a more stringent criterion because the intersection of confidence intervals tends to be low for selected probes. Moreover, the ratio between negative and positive issues accounts for another criterion. Each probe could theoretically be used as a unigenic predictor. The *α *parameter that we introduce to account for positive and negative outcomes can be adjusted to favor sensitivity or specificity of the multigenic predictor. In the case of a predictor based on a majority vote, the *α *parameter should be 0.5 to maintain equity.

Ideally, these probe selection methods could be combined to identify most relevant probes because these methods each take advantage of particular strengths in probe selection [12]. Additional studies should be carried out on multiple datasets to investigate complementary methods. The discrepancies between microarray data publicly available from pharmacogenomic programs (different platforms, different regimens, different methods of response assessment) precluded any possibility of additional validation. In further studies, we plan to test other prediction problems such as molecular classification or survival issue.

## Conclusion

In this study, we propose a valuation function that assigns the highest values to probes that correctly predict cases across the whole learning set, such that each probe not only successfully predict a large number of cases, but also predicts PCR and NoPCR cases in approximately the same ratio as was seen in the whole set. In addition to improving the prediction of patient response to breast cancer preoperative chemotherapy, our approach has made it possible to classify probes as bi-informative and mono-informative.

## Methods

### Patients

The clinical trial was conducted at the Nellie B. Connally Breast Center of The University of Texas M.D. Anderson Cancer Center. Patients with stage I-III breast cancer requiring neoadjuvant chemotherapy were asked to undergo single-pass, fine-needle aspiration (FNA) of the primary breast tumor or ipsilateral axillary metastasis before starting chemotherapy as part of an ongoing pharmacogenomic marker discovery program [30]. Neoadjuvant chemotherapy consisted of weekly paclitaxel administration, followed by chemotherapy with 5-fluorouracil, doxorubicin, and cyclophosphamide (T/FAC). Gene expression profiling was performed using oligonucleotide microarrays (Affymetrix U133A) on FNA specimens taken prior to treatment.

Patient cases were separated into a learning set (82 cases) and three validation/test sets (51, 147 and 50 patients). Characteristics of patients [see Additional file 8] in the first validation set were similar to those of learning set patients, and the two patient groups received similar chemotherapy regimens. Patients in the second validation set received a similar chemotherapy regimen as the learning set patients, but they showed different characteristics; for example, few patients in the second validation set showed HER2 amplification, (these patients received trastuzumab and were not included in the pharmacogenomic study). The third validation set comprised patients who had been treated at Gustave Roussy Institute, who had preoperative biopsies rather than FNA, and who underwent an anthracycline-based chemotherapy regimen without taxanes. These latter patients were matched pairs of patients with PCR and residual macroscopic disease.

At the completion of neoadjuvant chemotherapy, all patients underwent surgical resection of the tumor bed, with negative margins. Pathologic complete response (PCR) was defined as no histopathologic evidence of any residual invasive cancer cells in the breast, whereas residual disease (NoPCR) was defined as presence of residual cancer cells after histopathologic study [31].

The low-level treatment of the microarray data was performed using dCHIP V1.3 [32] to generate probe level intensities. This program normalizes all arrays to one standard array that represents a chip of median overall intensity. This reference chip and the normalization procedure are available online at [33]. Normalized gene expression values were transformed to the log_10 _scale for analysis [1].

### Methods

Consider a hypothetical ideal probe set s* which would be accurate enough for classifying the patients of the learning set into PCR and NoPCR groups. Knowing the expression level of this probe set in each of the patients would be enough to predict response to chemotherapy. Hence, the interval of expression levels *I*_*p*_(s*) contains the expression levels of the PCR patients in the learning set, while the interval *I*_*n*_(s*) contains the expression levels of the NoPCR cases of the learning set. Since the probe set s* is supposed to classify all the learning cases, these two intervals are disjoints, *I*_*p*_(s*) ∩ *I*_*n*_(s*) = ∅ ; otherwise at least one treatment outcome could not be predicted for learning-set cases from the observation of the ideal probe set's expression level. Given these definitions, any patient in the learning set should belong to one or the other interval; i.e. the expression level of any PCR learning case would be in the interval *I*_*p*_(s*) and that of any NoPCR one would be in the interval *I*_*n*_(s*).

#### Minimum sets of expression levels of the actual probe sets

For actual probe sets, the intervals of expression levels are not disjoint for PCR and NoPCR learning cases, and the expression levels are blurred by noise. Therefore, we decided to attach two minimum sets of expression levels to any probe set *s*. These sets, *E*_*p*_*(s) *and *E*_*n*_*(s)*, were computed from the learning set^1^.

#### Intervals of PCR and NoPCR expression levels

Let *m*_*p*_*(s) *and *sd*_*p*_*(s) *be the mean and standard deviation of the expression levels of probe set *s *for the PCR learning cases. The interval of 'PCR expression levels' of the probe set *s*, denoted *I*_*p*_*(s)*, is that of length *2 × sd*_*p*_*(s)*, centered on the mean *m*_*p*_*(s)*:

*I*_*p*_*(s) *= [*m*_*p*_*(s) *- *sd*_*p*_*(s)*, *m*_*p*_*(s) *+ *sd*_*p*_*(s)*]

In the same way, let *m*_*n*_*(s) *and *sd*_*n*_*(s) *be the mean and standard deviation of the expression levels of probe set *s *for the subset of NoPCR learning cases. The interval of 'NoPCR expressions' of the probe sets *s *is:

*I*_*n*_*(s) *= [*m*_*n*_*(s) *- *sd*_*n*_*(s)*, *m*_*n*_*(s) *+ *sd*_*n*_*(s)*]

#### Minimum sets of PCR and NoPCR expression levels

Since the intervals of expression levels *I*_*p*_*(s) *and *I*_*n*_*(s) *are not disjoint in general, we defined the minimum set of the PCR expression levels of the probe set *s*, denoted *E*_*p*_*(s)*, as the interval of PCR expressions *I*_*p*_*(s) *minus its intersection with the interval *I*_*n*_*(s)*. Conversely, we defined the minimum set of NoPCR expression levels, denoted *E*_*n*_*(s)*, as the interval of NoPCR expression levels *I*_*n*_*(s) *minus its intersection with the interval *I*_*p*_*(s)*:

• minimum set of PCR expression levels of the probe set *s*:

*E*_*p*_*(s) *= *I*_*p*_*(s) *\ (*I*_*p*_*(s) *∩ *I*_*n*_*(s)*)

• minimum set of NoPCR expression levels of the probe set *s*:

*E*_*n*_*(s) *= *I*_*n*_*(s) *\ (*I*_*p*_*(s) *∩ *I*_*n*_*(s)*)

#### Discrete prediction of a probe set

We define the prediction of a single probe set as a discrete value taken in the set {pcr, nopcr, unspecified} as follows: if patient *p *belongs to the learning set and the expression level of the probe set *s *for this patient *p *is *e(s, p)*, then the discrete prediction of the single probe set *s *is *pcr *if the expression level is in the minimum set of PCR expression levels: *e(s, p) *∈ *E*_*p*_*(s)*. On the other hand, the prediction is *nopcr *if the expression level is in the minimum set of NoPCR expression levels: *e(s, p) *∈ *E*_*n*_*(s)*. If neither of these cases obtains, the prediction is *unspecified*. A pcr prediction is correct when the learning case is a PCR case, and the same is true for a nopcr prediction of a NoPCR case.

#### Valuation of a probe set

From the definition of the *pcr *and *nopcr *predictions of a probe set, we define its valuation *v(s)*. Let *p(s) *be the number of PCR learning cases correctly predicted by the probe set, let *n(s) *be the number of NoPCR learning cases correctly predicted by the probe set, and let *P *and *N *be the respective numbers of PCR and NoPCR learning cases. The probe sets' valuation function *v(s)*, whose values are in the real interval [0, 1], is:

$$\text{v(s) }=0.5\times \left(\frac{p(s)}{P}+\frac{n(s)}{N}\right)$$

(The coefficient 0.5 serves only to ensure that the values of the function lie within the unit interval.)

This function assigns a value of 1 to a hypothetical ideal probe set and a value of 0 to a non-informative one. This very simple function takes into account the proportions of PCR and the proportion of NoPCR learning cases correctly predicted by the probe set rather than simply the total proportion of correctly predicted learning cases. The reason for this choice is that the proportion of correctly predicted learning cases, regardless of their classes, would obviously be biased by the unequal numbers of PCR and NoPCR cases in the learning set. It is worth noting that the valuation function *v(s) *does not take into account either incorrect or unspecified predictions of the probe set.

#### Validation of reproducibility

The reproducibility and robustness of the present method was tested in 30 replicate experiments when the same RNA was hybridized twice several months apart in two different laboratories [34]. Valuation functions of the 30 highest-ranking probe sets were correlated in replicate experiments.

#### Multigenic predictors

We tested several multigenic predictors. First, we developed a majority vote predictor that could take advantage of the discrete predictions of probe sets. Hence, for any patient, the prediction of a probe set was 'pcr' if the expression level of this probe set for this patient was in its minimum set of PCR expression levels, the prediction was 'nopcr' if the expression level was in its minimum set of NoPCR expression levels, or the prediction was unspecified. In the first two cases, the prediction of a probe set can be either correct or incorrect.

We have defined *k *probe set predictor as, on the one hand, the set *S*_*k *_composed of the *k *top-ranked probe sets and, on the other hand, a decision criterion which was the majority decision. Let *p *be a patient case, let *pcr(k, p) *be the number of probe sets of set *S*_*k *_whose predictions are *pcr *for this patient, and let *nopcr(k, p) *be the corresponding number of nopcr predictions. The *k* probe set predictor then indicates the following responses for this patient *p*:

• if *pcr(k, p) *> *nopcr(k, p) *then PCR;

• if *pcr(k, p) *<*nopcr(k, p) *then NoPCR;

• if *pcr(k, p) *= *nopcr(k, p) *then UNSPECIFED.

When evaluating the performances of the predictor, a false negative is a PCR patient case predicted to be NoPCR or UNSPECIFIED; a false positive is a NoPCR patient case predicted to be PCR or UNSPECIFIED.

#### Diagonal linear discriminant analysis (DLDA), and nearest centroid predictors

We evaluated the performance of the probes in the training dataset by leave-one-out cross validation (LOOCV) and by k-fold validation, in which case we used k = 3 and performed 1000 permutations to determine p-values. These multigenic predictor experiments were performed using BRB-Arraytools [35].

#### External validation

We evaluated the performance of a DLDA multivariate predictor in independent datasets. The 30 probe sets with highest t-test p-values or the 30 probe sets scoring the highest in the valuation function were used in two multivariate predictors constructed using a DLDA machine learning algorithm. The DLDA prediction model was tested on the three independent validation sets and their performance was compared using receiver-operating characteristic (ROC) curve analysis and performance metrics such as sensitivity. Classifier performance (discrimination) on the validation data was assessed using the area under the ROC curve (AUC). The ROC curve is a graphical display of the false-positive rate and the true-positive rate under multiple classification rules. The ROC curve arises when a continuous predictor value is calculated for each subject over a broad range of thresholds. A case is called test-positive (predicted to have PCR) if the threshold is above a defined value. The total area under the ROC curve is a summary measure of the test's ability to correctly classify those with and without the outcome of interest. An AUC of 1 represents a perfect test; an AUC of 0.5 represents a test no better than random prediction.

## Abbreviations

PCR: pathologic complete response; NoPCR: residual disease; DLDA: Diagonal linear discriminant analysis; LOOCV: leave-one-out cross validation; *v*: valuation function; FNA: fine-needle aspiration; ROC: receiver-operating characteristic curve; AUC: area under the ROC curve; FN: false negative; FP: false positive

## Authors' contributions

RN defined the method and the valuation function. He wrote the computer programs except those for computing the minimum sets of expression levels without noise filtering, and the cross validation. He established the upper bound of the p-value of the predictors in cooperation with RI. RI is the expert of the group in Paris for microarrays issues. He contributed to the reflections leading to the method and to establish the upper bound of the p-value of the predictors. EGH, BC, and PG contributed to the study in the frame of two student projets at the master level. They wrote the computer programs for the minimum sets of expression levels without noise filtering and the cross validation. They were involved in the early stage of this study which was concerned by the use of neural networks for predicting the responses to chemotherapy. KY, CC, and FA provided additional data sets and contributed actively to test the methods in the validation sets. KY took part in the comparison of the model with the linear discriminant analysis classifier. LP supervised the clinical trial from which the data were collected. He made fruitful comments and suggestions for improving the quality of this article and suggested the comparisons against predictors designed on random labeled learning sets. RR is the expert of the group in Paris for the biological and medical issues. He took part in the clinical trial. He contributed to the reflections leading to the method and established the comparison of the model with the linear discriminant analysis classifier and nearest centroid method. All authors read and approved the final manuscript.

## Supplementary Material

Additional file 1 Figure – Expression levels of a PCR probe set, probe *s *= 213033_s_at of gene NFIB, for the 82 cases of the learning set. The data provided represent a PCR probe set. Click here for file

Additional file 2 Figure – Expression levels of a NoPCR probe set, probe *s *= *s *= 203928_x_at of gene MAPT, for the 82 cases of the learning set. The data provided represent a NoPCR probe set. Click here for file

Additional file 3 Figure – Discriminations of the two DLDA classifiers (30 probes with the highest valuation functions, and 30 probe sets showing the highest p-values (t-test)) in the independent test set 3. The data provided represent the performance metrics obtained in test set 3. Click here for file

Additional file 4 Appendix – P-value of the predictors. The data provide the method used to calculate the p-values of the 27, 29, and 30 probe set predictors based on the null hypothesis of a predictor composed of random probe sets. Click here for file

Additional file 5 Table – Performance metrics of a multigene majority vote predictor (weighted valuation functions) for *α *∈ {0, 0.1,...,1.0}. The data provided represent a family of valuation functions, *v*_*α*_*(s)*, parameterized by the real number alpha, *α *∈ [0, 1]. Click here for file

Additional file 6 Table – Ratios of pcr to nopcr predictions for the weighted valuation functions; P(*α*), N(*α*): total numbers of pcr and nopcr predictions of the top 30 probes in the ranking v_*α*_(s); R(*α*) = P(*α*)/N(*α*). The data provided represent the results obtained by parameterization of the valuation function by the real number alpha, *α *∈ [0, 1]. Click here for file

Additional file 7 Figure – Sets top 30 probes for the weighted valuation functions. Underlined probes: mono-informative probes (either PCR or NoPCR probes). The data provided represent the top 30 probes obtained by parameterization of the valuation function by the real number alpha, *α *∈ [0, 1]. Click here for file

Additional file 8 Table – Patients characteristics. The data provided give the patient characteristics for the 4 cohorts of the study. Click here for file
