# Supplementary figures and images for: Prediction of the outcome of preoperative chemotherapy in breast cancer using DNA probes that provide information on both complete and incomplete responses
Source: BMC Bioinformatics. 2008 Mar 15;9:149. doi: 10.1186/1471-2105-9-149 (PMC2292140; doi:10.1186/1471-2105-9-149)

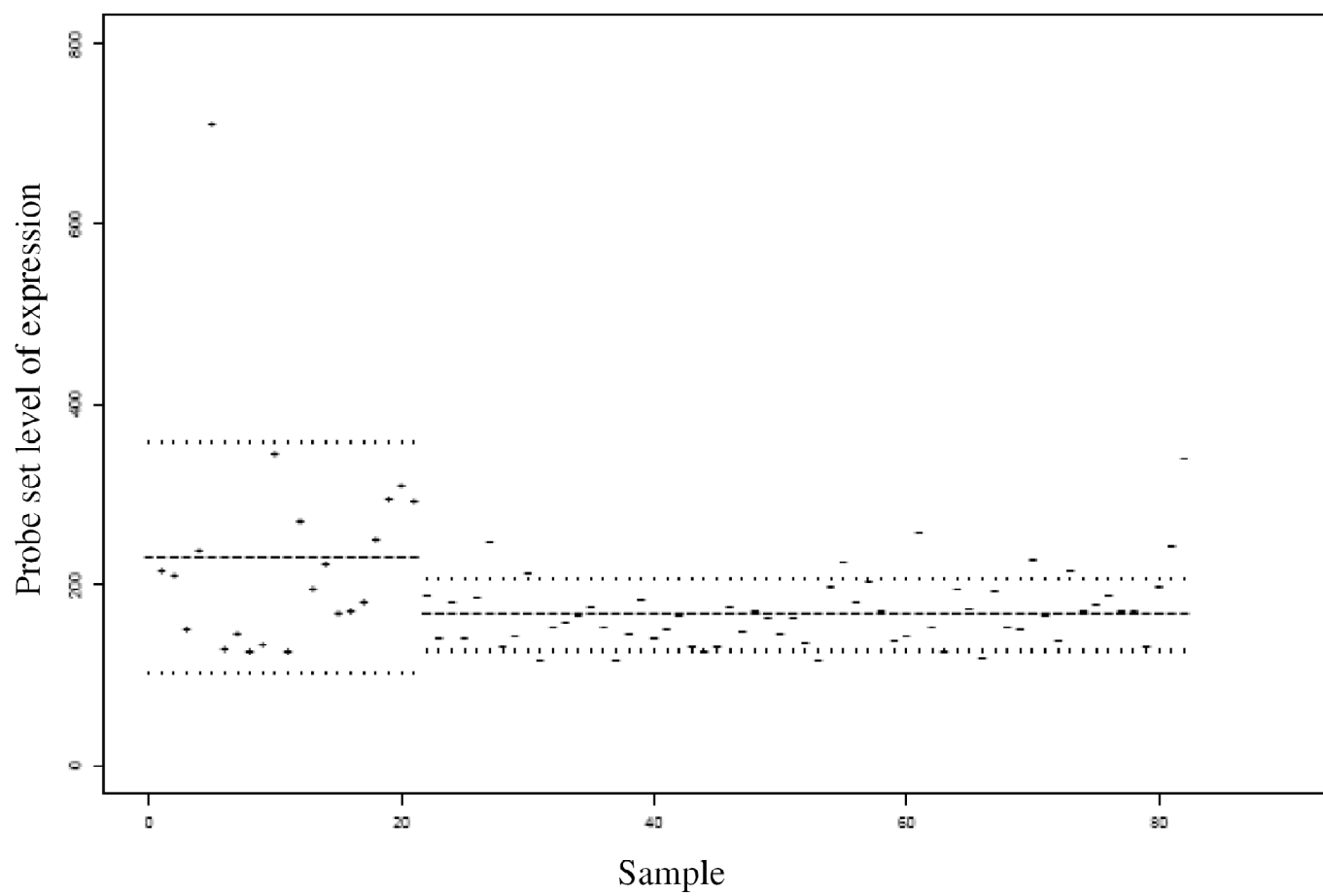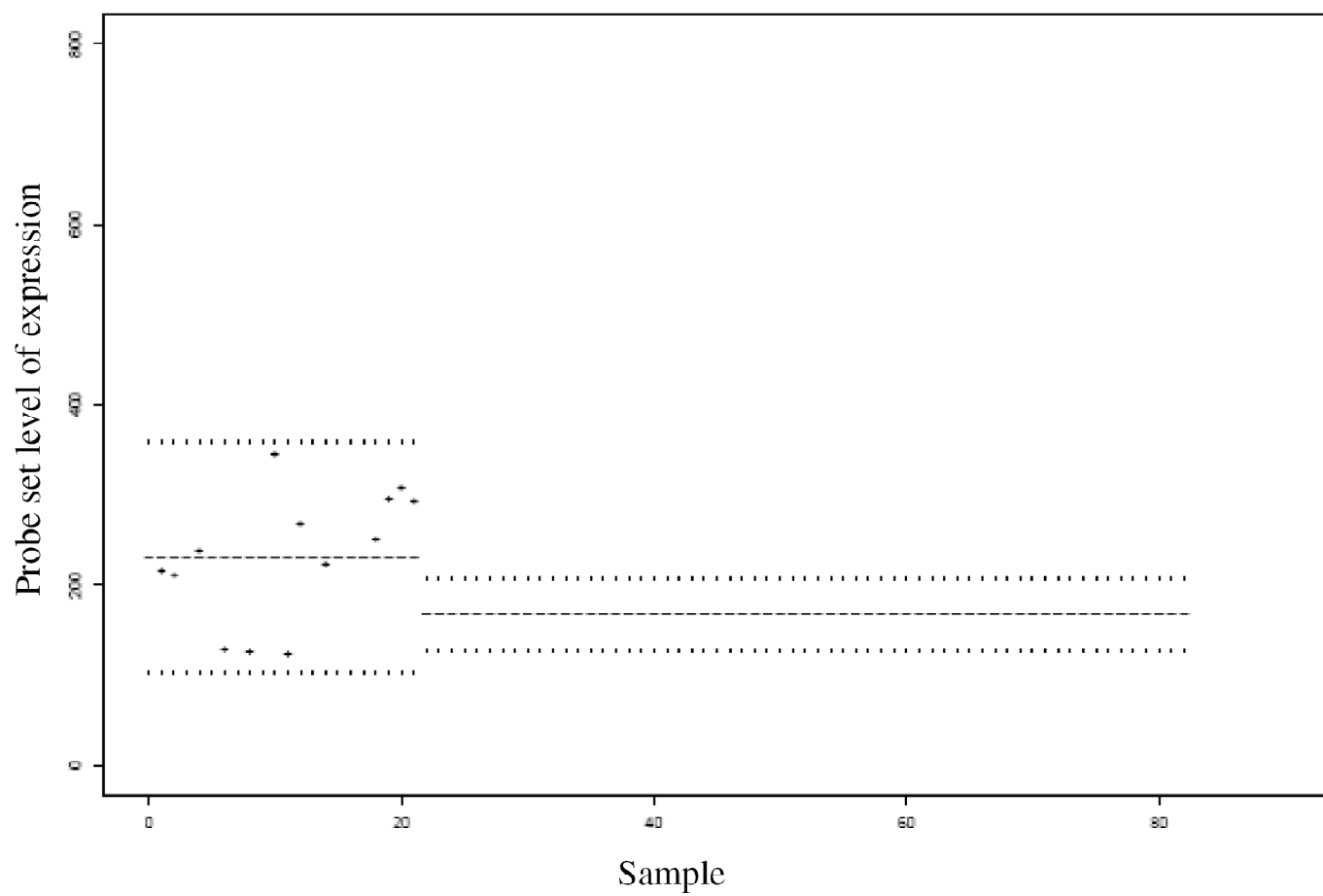

Supplement: Additional file 1 — Figure – Expression levels of a PCR probe set, probe s = 213033_s_at of gene NFIB, for the 82 cases of the learning set. The data provided represent a PCR probe set. [file 1471-2105-9-149-S1.pdf]

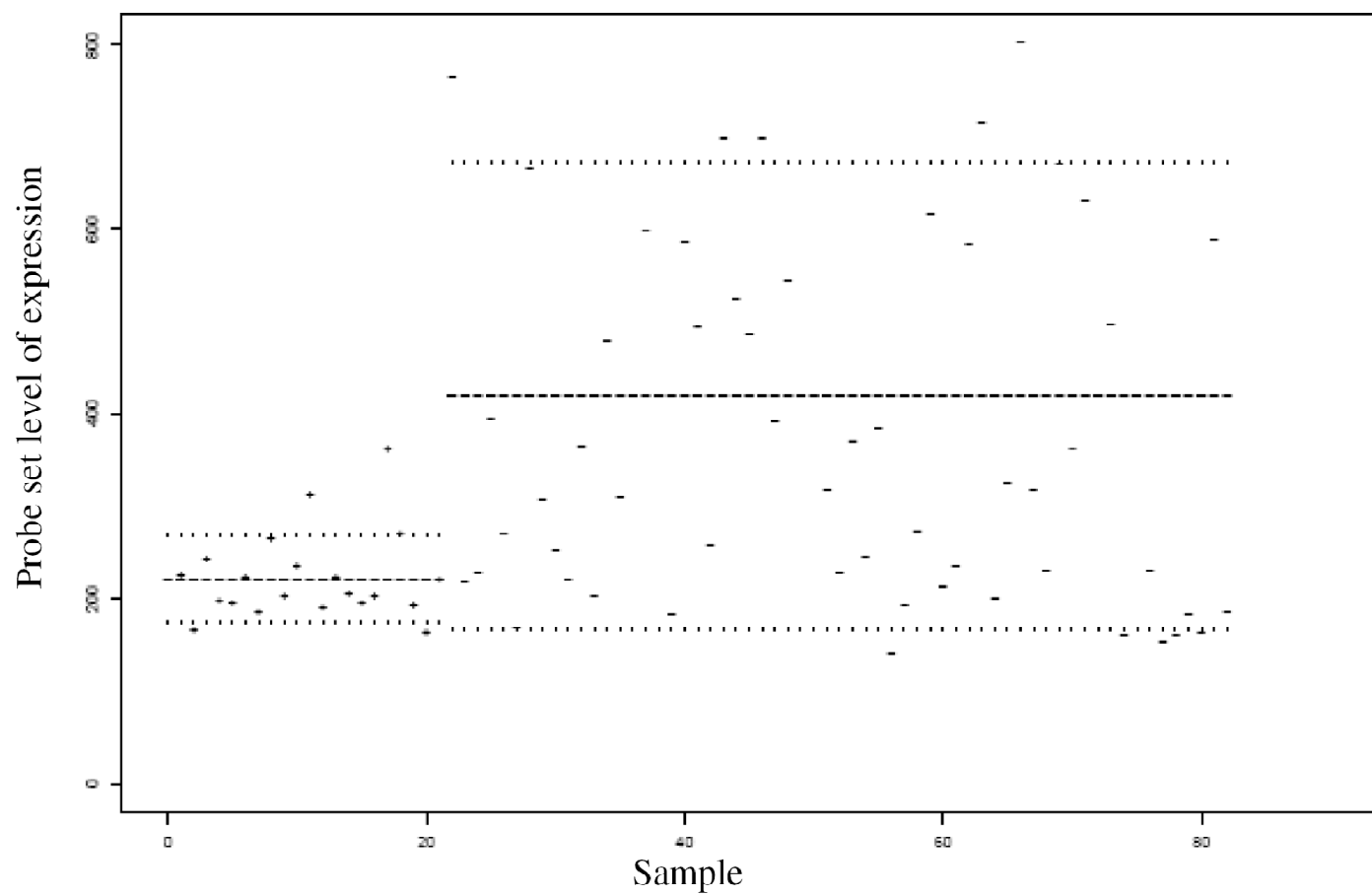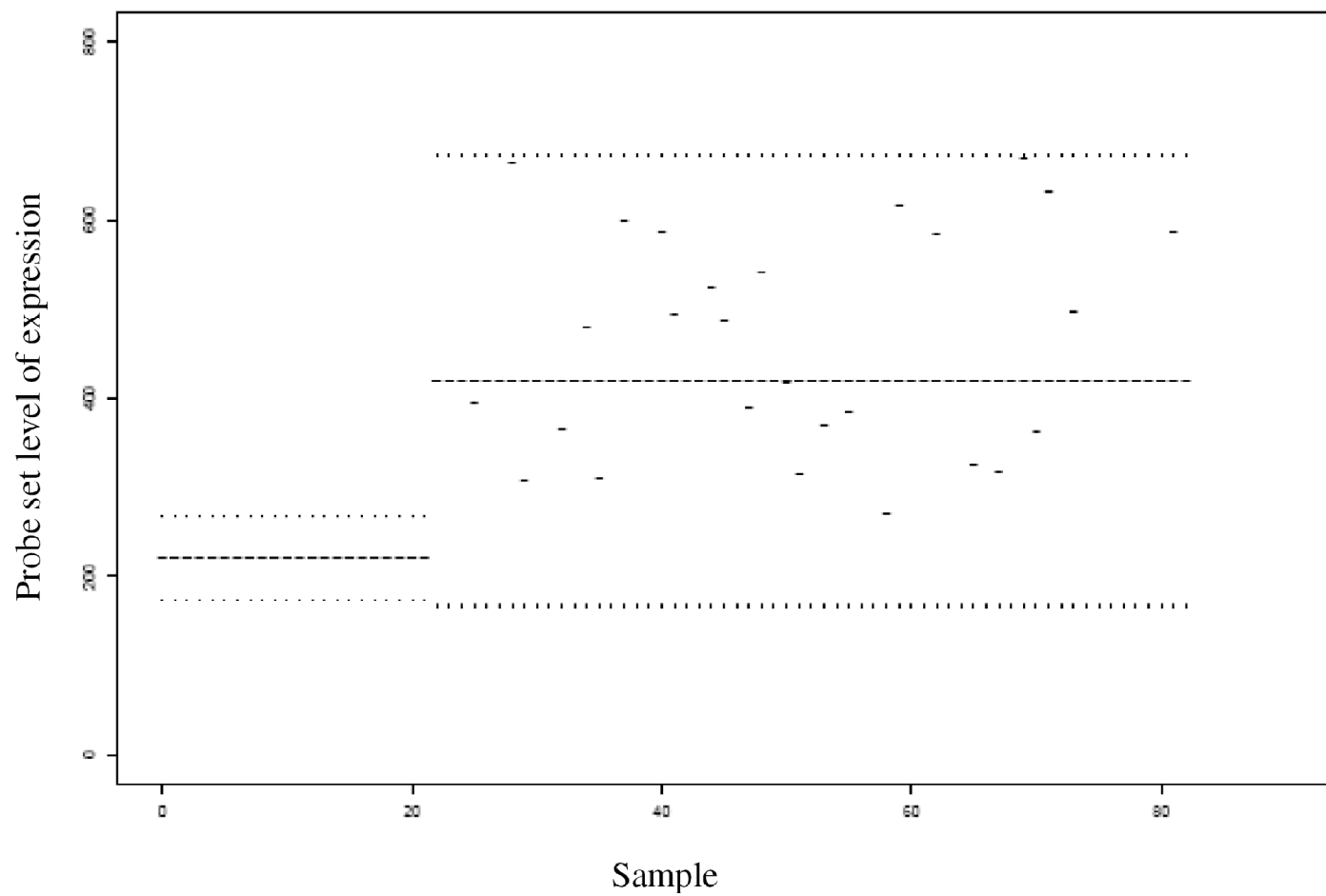

Supplement: Additional file 2 — Figure – Expression levels of a NoPCR probe set, probe s = s = 203928_x_at of gene MAPT, for the 82 cases of the learning set. The data provided represent a NoPCR probe set. [file 1471-2105-9-149-S2.pdf]

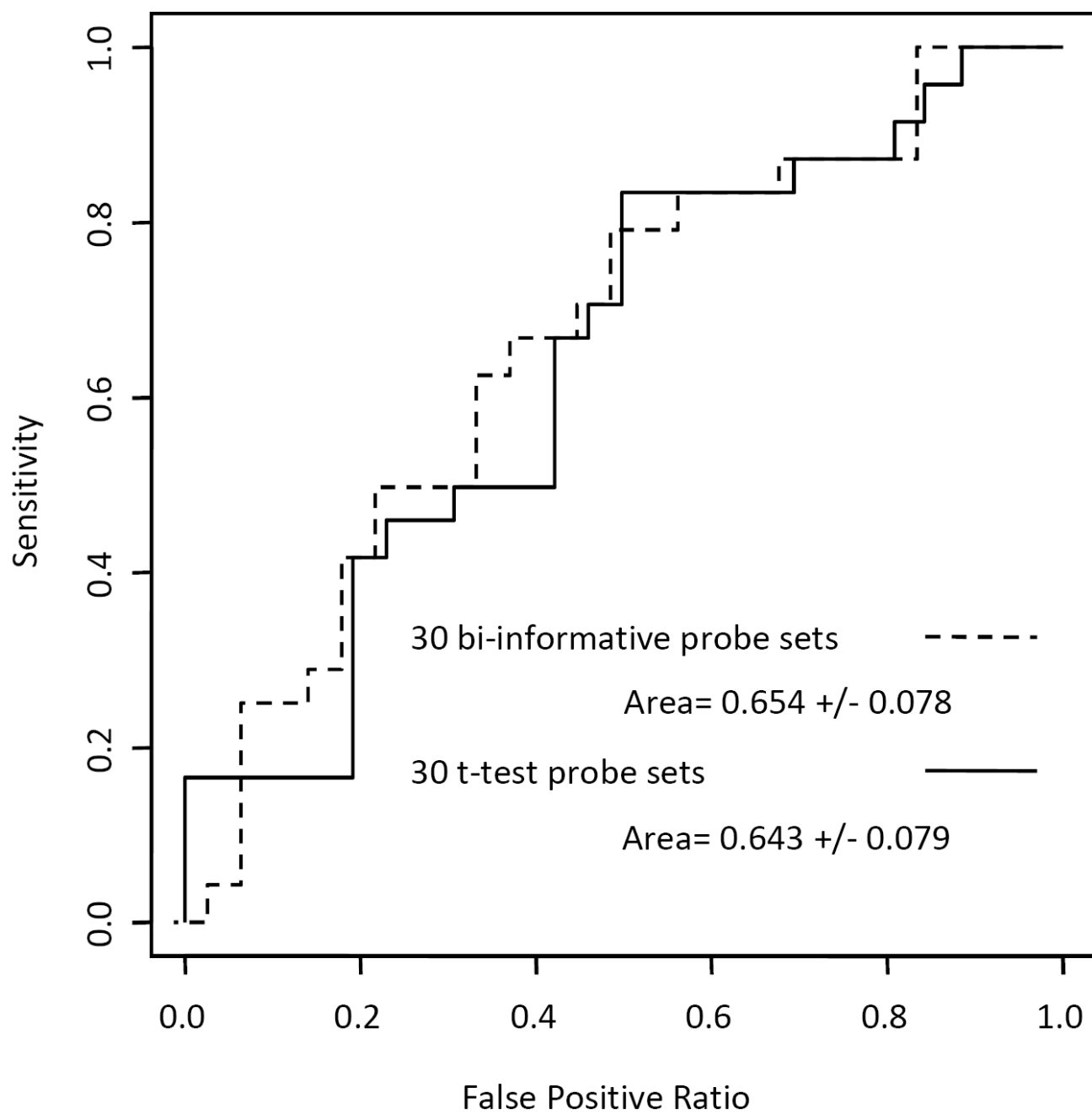

Supplement: Additional file 3 — Figure – Discriminations of the two DLDA classifiers (30 probes with the highest valuation functions, and 30 probe sets showing the highest p-values (t-test)) in the independent test set 3. The data provided represent the performance metrics obtained in test set 3. [file 1471-2105-9-149-S3.pdf]
