# Supplementary material for: Prediction of the outcome of preoperative chemotherapy in breast cancer using DNA probes that provide information on both complete and incomplete responses
Source: BMC Bioinformatics. 2008 Mar 15;9:149. doi: 10.1186/1471-2105-9-149 (PMC2292140; doi:10.1186/1471-2105-9-149)
Supplement: Additional file 4 — Appendix – P-value of the predictors. The data provide the method used to calculate the p-values of the 27, 29, and 30 probe set predictors based on the null hypothesis of a predictor composed of random probe sets. [file 1471-2105-9-149-S4.doc]

**Appendix : P-value of the predictors**

We establish an upper bound for the P-value of the 30-probes predictor. This predictor achieved the best performance of 12 PCR correct predictions and 32 NoPCR correct predictions out of 13 PCR and 38 NoPCR validation cases. Its p-value is less than 1.12×10^-12^. This upper bound also holds for the 27 and the 29-probes predictors that achieved the performance of the 30-probes predictor.

Recall the probe’s individual prediction of probe *s* for a patient *p* is *pcr* if the expression level *e(p,s)* of the probe for this patient is in the probe’s minimum set of PCR expression levels: *e(s,p)* ∈ *E_p_(s)* (see “Methods”). In this case, the probe predicts that the outcome of the treatment will be a pathologic complete response.

Respectively, the probe’s individual prediction is *nopcr* if the expression level is in the probe’s minimum set of NoPCR expression levels: *e(s,p)* ∈ *E_n_(s)*. In this case, the probe predicts that the patient will not have a pathologic complete resopnse. Otherwise, the probe’s individual prediction is *unspecified*. In this case, the probe cannot predict the outcome of the treatment.

**Null hypothethis.**

On the validation set, let us denote by *p(p)*, *p(n)* and *p(u)* the respective probabilities that the prediction of a probe chosen at random is pcr, nopcr or unspecified.

Let us define a “N-random-probes predictor” as a predictor made out of *N* random probes, and whose prediction criterion is the strict majority vote: if the number of pcr probe predictions is strictly greater than that of the nopcr ones then the patient is predicted as PCR, and if this number is strictly lower, the patient is predicted as NoPCR. Our null hypothesis is that the prediction of the treatment outcome is made by a N-random-probes predictor.

As an example, in section “Performances of the k-probes predictors”, we have seen that the 30-probes predictor that we propose predicted the outcome of 44 patient cases out of the 51 validation cases: 12 PCR cases out of 13 and 32 NoPCR cases out of 38. According to our null hypothesis, the p-value of this predictor is the probability of the 30-random-probes predictor to have a better performance.

**Probabilities of predictions for a randomly chosen probe.**

The set of validation patient cases contained a total of 51 cases. Since the total number of probes was 22 283, the total number of predictions was

*P_t_*=22283 × 51=1136433. On this validation set, the total number of *pcr* probe predictions was *P_p_* =12 701 and that of *nopcr* predictions was *P_n_* =92 972. Hence, the total number of *unspecified* predictions was *P_u_* = *P_t_* – *P_p_* - *P_n_* =1030760 and the probabilities of probe predictions were:

- *p(p)* = *P_p_* / *P_t_* = 0.0111762;
- *p(n)* = *P_p_* / *P_t_* = 0.0818104;
- *p(u)* = *P_u_* / *P_t_* = 0.907013.

**Probabilities of predictions for N-random-probes predictors.**

Knowing the probabilities of the probe predictions, one can establish the probabilty *P(TIE)* of a N-random-probes predictor to face an equal number of *pcr* and *nopcr* probe predictions, i.e.

a tie. This occurs when *N-2k* probe predictions are *unspecified* and *k* probe predictions are *pcr* and *k* probe predictions are *nopcr*. There are $C_{N}^{N-2k}$ different choices of *N-2k* probes among *N* probes. These *N-2k* probes being chosen, there are $C_{2k}^{k}$ different choices left of having *k* probes predicting a *pcr* outcome among the *2k* remaining probes. These *k* probes being fixed, the *k* remaining ones have to predict a *nopcr* outcome. Hence, the probability *P(TIE)* is:

$$P(TIE)=\sum_{k=0}^{N/2} [C_{N}^{N-2k}\times p(u)^{N-2k}]\times[C_{2k}^{k}\times(p(p)\times p(n))^{k}]$$

Numerically, we have *P(TIE)*=0.117017.

Knowing the probability of a tie, we have an obvious upper bound of the probability *P(PCR)* of the N-random-probes predictor to predict that patient *p* is PCR:

*P(PCR)*=1-(*P(TIE)*+*P(NoPCR)*)<1-*P(TIE)*

The same upper bound holds for the NoPCR prediction:

*P(NoPCR)*=1-(*P(TIE)*+*P(PCR)*) < 1-*P(TIE)*

Numerically, we have *P(PCR)*<0.882983 and the same for *P(NoPCR)*.

**P-value of the N-probes predictors.**

The predictions of patients’ treatment outcomes being independant, the probability *P(K,K’)* of the event “*K* patient cases are predicted PCR and *K’* patients are predicted NoPCR” is

*P(K,K’)*=*P(PCR)^K^* × *P(NoPCR)^K’^* < *P(PCR)^K+K’^* = 0.882983*^K+K’^*

The 30-probes predictor of section ``Performances of the k-probes predictors’’ predicted *K*=12 PCR outcomes and *K’*=32 NoPCR outcome. For this predictor, one has *P*(12,32)=0.004187001 < 0.005. From this last probability we can give an upper bound of the probability *P**(12,32) of the event “the predictions of a random predictor are correct for 12 PCR cases and 32 NoPCR cases out of 51 cases”. There are $C_{51}^{12}$ subsets of 12 patients among the 51 patients of the validation set, and $C_{39}^{32}$ subsets of 32 patients among the remaining 51-12=39 patients. So, there are $C_{51}^{12}$ × $C_{39}^{32}$ different disjoint subsets of 12 and 32 patients of the validation set. Furthermore, the number of subsets of 12 PCR patients is $C_{13}^{12}$ and that of 32 NoPCR patients is $C_{38}^{32}$. Hence an upper bound of the probability *P*(12,32) is

$$P*(12,32)<0.005\times\frac{C_{13}^{12}\times C_{38}^{32}}{C_{51}^{12}\times C_{39}^{32}}=7.34893\times10^{-14}<8\times10^{-14}$$

Since *PV* is the probability of a 30-random-probes predictor to outperform the correct predictions of 12 PCR out of 13 and 32 NoPCR cases out of 38, we have

*PV*=*P**(12,32)+*P**(13,32)+ ... *P**(12,38)+ *P**(13,38)

We can now give an upper bound for the p-value *PV* of the 30-probes predictor:

*PV* < 14 × *P**(12,32) < 14 × 8 ×10^-14^ = 1.12 × 10^-12^

We saw in section “Performances of the k-probes predictors” that the 27-probes predictor and the 29-probes predictors had the same performances than the 30-probes predictor. The upper bound of the 30-probes predictor is also an upper bound for that of the 27 and 29-probes predictors since it only depends on the number *N* of probes through the probabilities *P(PCR)* and *P(NoPCR)*, themselves decreasing in *N*.

**Random labeling of the learning cases.**

Here we consider learning sets composed of randomly labeled cases, and the performances of the *k*-probes predictors made of probes whose valuations were computed on such random learning sets.

The initial learning set *L* was composed of 21 PCR and 61 NoPCR learning cases. Let *S_p_* and *S_n_* be these two subsets. We randomly dispatched the cases of the learning set *L* into two subsets *S’_p_* and *S’_n_* composed of 21 and 61 cases and we respectively labeled their cases PCR and NoPCR, regardless of the actual outcome of the treatment. Because of the initial distribution of PCR and NoPCR learning cases (1/3 and 2/3 of the total), on average 1/3 of the cases of the subset *S’_p_* were actual PCR cases and 2/3 of the cases of the subset *S’_n_* were actual NoPCR cases.

For any probe *s* let *m_p_(s)* be the mean of its expression levels computed on the subset *S_p_* and *m’_p_(s)* be the mean computed on the subset *S’_p_*, and let *m_n_(s)* and *m’_n_(s)* be the means computed on subsets *S_n_* and *S’_n_*.

**Probes’ ranking.**

For sake of clarity, in the following of this section we consider a typical randomly labeled set *L’* whose subset *S’_p_* is composed of 5 actual PCR cases and 16 actual NoPCR cases chosen at random, therefore its subset *S’_n_* is composed of 16 actual PCR and 45 actual NoPCR cases. Let *R* be the ranking of the probes for the initial learning set *L*, *R’* their ranking for the randomly labeled learning set *L’*, and let *P_30_* and *P’_30_* be the first 30 probes in the respective rankings *R* and *R’*. The minimum rank of the probes of *P_30_* in the ranking *R’* was 1412 and the mean rank was 8878. Conversely, the minimum rank of the probes of *P’_30_* in the ranking *R* was 1005 and the mean rank was 8045. This shows that randomizing the labels of the learning cases deeply affected the values of the probes.

**Mean numbers of probes predictions.**

For the initial learning set *L*, the total number of *pcr* and *nopcr* probes’ individual predictions was 203799 (22925 pcr and 180874 nopcr predictions). For the randomly labeled learning set *L’* the total number was 125404 (26500 pcr and 98904 nopcr predictions). Hence, the initial learning set *L* brought 62% more correct predictions of the learning cases. This shows that random case labeling deeply affected the probability of a patient to express a probe in one of its two minimum sets of expression levels.

**Ratios of pcr to nopcr predictions and performances the k-probes predictors.**

The total ratio of pcr to nopcr predictions was 12.67% for the learning set *L* and twice for the random learning set *L’* (26.79%). With such an increase of this ratio in favor of the random learning set *L’*, one could expect the predictors with probes’ valuations computed on learning set *L’* to be more sensitive that the predictors with probes’ valuations computed on the learning set *L*. But this was not the case: almost all the cases were predicted to be NoPCR. None of the predictors with probes’ valuations computed on *L’* do predicted the outcome of more than 2 PCR validation cases, and the predictors made of more than 25 probes predicted no less than 36 NoPCR cases (out of the 13 PCR and 38 NoPCR validation cases). The best performance was for predictors with *k*-probes, 41 ≤ *k* ≤ 45: accuracy=39/51=0.76, sensitivity=2/13=0.15, specificity=37/38=0.97, PPV=2/3=0.66, NPV=37/49=0.76. These predictors had low sensitivity and high specificity.

For understanding this result, recall that only 1/3 of the cases in *S’_p_* were actual PCR cases and 2/3 of the cases in *S’_n_* were actual NoPCR cases. If a probe is that of a gene not concerned by the response to the chemotherapy, the means and standard deviations of its expression levels have close values on the initial subsets *S_p_* and *S_n_*. These values will remain close on the random subsets *S’_p_* and *S’_n_*. Conversely, if the gene is concerned by the response to the chemotherapy, the means are significantly different on sets *S_p_* and *S_n_*. We will suppose that the standard deviations are close on these two sets, which is the case for the vast majority of bi-informative probes. Hence, since only 1/3 of the cases of the set *S’_p_* are actual PCR cases, the means of the expression levels will differ a lot between sets *S_p_* and *S’_p_*. Concerning the sets *S_n_* and *S’_n_*, their means will also be different but to a lesser extend because 2/3 of the cases of the random set *S’_n_* are actual NoPCR cases. Then, the probability of a probe to predict a NoPCR case as nopcr is higher than the probability of a probe to predict a PCR case as pcr. It follows that the predictors built on such randomly labeled learning sets of cases predicted almost none of the PCR validation cases and almost all the NoPCR ones.
