# Supplementary material for: Prediction of the outcome of preoperative chemotherapy in breast cancer using DNA probes that provide information on both complete and incomplete responses
Source: BMC Bioinformatics. 2008 Mar 15;9:149. doi: 10.1186/1471-2105-9-149 (PMC2292140; doi:10.1186/1471-2105-9-149)
Supplement: Additional file 5 — Table – Performance metrics of a multigene majority vote predictor (weighted valuation functions) for α ∈ {0, 0.1,...,1.0}. The data provided represent a family of valuation functions, vα(s), parameterized by the real number alpha, α ∈ [0, 1]. [file 1471-2105-9-149-S5.doc]

Supplemental Table 1: Performance metrics of a multigene majority vote predictor (weighted valuation functions) for *α*∈{0, 0.1, ..., 1.0}

| **α** | **Acc** | **Se** | **Sp** | **TP** | **TN** | **FN** | **FP** |
| --- | --- | --- | --- | --- | --- | --- | --- |
| **0.0** | 0.75 | 0 | 1 | 0 | 38 | 13 | 0 |
| **0.1** | 0.75 | 0 | 1 | 0 | 38 | 13 | 0 |
| **0.2** | 0.75 | 0 | 1 | 0 | 38 | 13 | 0 |
| **0.3** | 0.73 | 0 | 0.97 | 0 | 37 | 13 | 1 |
| **0.4** | 0.86 | 0.77 | 0.89 | 10 | 34 | 3 | 4 |
| **0.5** | 0.86 | 0.92 | 0.84 | 12 | 32 | 1 | 6 |
| **0.6** | 0.84 | 0.85 | 0.84 | 11 | 32 | 2 | 6 |
| **0.7** | 0.82 | 0.85 | 0.68 | 10 | 32 | 3 | 6 |
| **0.8** | 0.73 | 0.85 | 0.68 | 11 | 26 | 2 | 12 |
| **0.9** | 0.67 | 0.85 | 0.61 | 11 | 23 | 2 | 15 |
| **1.0** | 0.49 | 1 | 0.32 | 13 | 12 | 0 | 26 |
