# Supplementary material for: Prediction of the outcome of preoperative chemotherapy in breast cancer using DNA probes that provide information on both complete and incomplete responses
Source: BMC Bioinformatics. 2008 Mar 15;9:149. doi: 10.1186/1471-2105-9-149 (PMC2292140; doi:10.1186/1471-2105-9-149)
Supplement: Additional file 6 — Table – Ratios of pcr to nopcr predictions for the weighted valuation functions; P(α), N(α): total numbers of pcr and nopcr predictions of the top 30 probes in the ranking vα(s); R(α) = P(α)/N(α). The data provided represent the results obtained by parameterization of the valuation function by the real number alpha, α ∈ [0, 1]. [file 1471-2105-9-149-S6.doc]

Supplemental Table 2. Ratios of pcr to nopcr predictions for the weighted valuation functions

P(α), N(α): total numbers of pcr and nopcr predictions of the top 30 probes in the ranking v_α_ (s);

R(α) = P(α)/N(α).

| **α** | **P(α)** | **N(α)** | **R(α)** |
| --- | --- | --- | --- |
| **0** | 40 | 1120 | 0.0357143 |
| **0.1** | 55 | 1119 | 0.049151 |
| **0.2** | 84 | 1106 | 0.0759494 |
| **0.3** | 152 | 1041 | 0.146013 |
| **0.4** | 247 | 897 | 0.275362 |
| **0.5** | 276 | 828 | 0.333333 |
| **0.6** | 295 | 765 | 0.385621 |
| **0.7** | 317 | 649 | 0.488444 |
| **0.8** | 334 | 489 | 0.683027 |
| **0.9** | 337 | 448 | 0.752232 |
| **1** | 337 | 332 | 1.01506 |
