# Supplementary material for: Prediction of the outcome of preoperative chemotherapy in breast cancer using DNA probes that provide information on both complete and incomplete responses
Source: BMC Bioinformatics. 2008 Mar 15;9:149. doi: 10.1186/1471-2105-9-149 (PMC2292140; doi:10.1186/1471-2105-9-149)
Supplement: Additional file 7 — Figure – Sets top 30 probes for the weighted valuation functions. Underlined probes: mono-informative probes (either PCR or NoPCR probes). The data provided represent the top 30 probes obtained by parameterization of the valuation function by the real number alpha, α ∈ [0, 1]. [file 1471-2105-9-149-S7.pdf]

$\alpha = 0$ (0 PCR probe, 24 NoPCR probes)<br>207067_s_at, 212745_s_at, 213134_x_at, 205548_s_at, 205326_at, 214519_s_at, 220252_x_at, 218468_s_at, 209016_s_at, 206496_at, 203682_s_at, 218254_s_at, 217399_s_at, 209773_s_at, 205645_at, 201266_at, 221483_s_at, 220801_s_at, 219287_at, 213001_at, 210983_s_at, 205768_s_at, 205278_at, 203699_s_at, 203287_at, 201405_s_at, 217770_at, 214843_s_at, 208795_s_at, 208781_x_at
$\alpha = 0.1$ (0 PCR probe, 22 NoPCR probes)<br>207067_s_at, 213134_x_at, 205548_s_at, 212745_s_at, 205326_at, 209016_s_at, 214519_s_at, 220252_x_at, 218468_s_at, 206496_at, 203682_s_at, 209773_s_at, 203287_at, 201755_at, 218254_s_at, 217399_s_at, 205645_at, 201266_at, 212207_at, 221483_s_at, 220801_s_at, 219287_at, 213001_at, 210983_s_at, 205768_s_at, 205278_at, 203699_s_at, 201405_s_at, 217770_at, 214843_s_at
$\alpha = 0.2$ (0 PCR probe, 19 NoPCR probes)<br>213134_x_at, 205548_s_at, 212745_s_at, 207067_s_at, 209016_s_at, 201755_at, 205326_at, 209604_s_at, 212207_at, 209773_s_at, 214519_s_at, 203287_at, 220252_x_at, 218468_s_at, 206496_at, 203682_s_at, 212660_at, 200891_s_at, 218254_s_at, 217399_s_at, 205645_at, 201266_at, 221483_s_at, 220801_s_at, 219287_at, 213001_at, 210983_s_at, 205768_s_at, 205278_at, 203699_s_at
$\alpha = 0.3$ (0 PCR probes, 11 NoPCR probes)<br>213134_x_at, 205548_s_at, 209604_s_at, 212745_s_at, 209016_s_at, 207067_s_at, 212207_at, 201755_at, 203287_at, 209603_at, 209773_s_at, 212660_at, 200891_s_at, 205326_at, 205339_at, 214519_s_at, 202200_s_at, 211302_s_at, 220252_x_at, 218468_s_at, 206496_at, 203682_s_at, 204825_at, 203226_s_at, 203009_at, 204862_s_at, 218254_s_at, 217399_s_at, 205645_at, 201266_at
$\alpha = 0.4$ (0 PCR probe, 1 NoPCR probes)<br>213134_x_at, 205548_s_at, 209604_s_at, 209016_s_at, 212207_at, 209603_at, 201755_at, 212745_s_at, 205339_at, 212660_at, 200891_s_at, 201826_s_at, 211302_s_at, 204862_s_at, 207067_s_at, 203287_at, 204825_at, 219051_x_at, 209773_s_at, 202200_s_at, 203226_s_at, 203009_at, 219044_at, 203693_s_at, 203139_at, 215867_x_at, 214164_x_at, 212046_x_at, 202392_s_at, 202370_s_at
$\alpha = 0.5$ (0 PCR probe, 0 NoPCR probe)<br>213134_x_at, 205548_s_at, 209604_s_at, 209603_at, 212207_at, 201826_s_at, 205339_at, 209016_s_at, 201755_at, 204862_s_at, 219051_x_at, 211302_s_at, 212660_at, 200891_s_at, 202392_s_at, 204825_at, 215867_x_at, 214164_x_at, 212046_x_at, 209602_s_at, 212745_s_at, 203139_at, 203226_s_at, 219044_at, 203693_s_at, 220016_at, 214383_x_at, 212721_at, 202200_s_at, 217028_at
$\alpha = 0.6$ (0 PCR probe, 0 NoPCR probe)<br>209604_s_at, 213134_x_at, 205548_s_at, 209603_at, 201826_s_at, 205339_at, 219051_x_at, 209602_s_at, 212207_at, 204862_s_at, 202392_s_at, 211302_s_at, 215867_x_at, 214164_x_at, 212046_x_at, 201755_at, 41660_at, 209016_s_at, 203139_at, 205425_at, 204825_at, 212660_at, 200891_s_at, 220016_at, 214383_x_at, 212721_at, 214058_at, 218065_s_at, 219044_at, 203693_s_at
$\alpha = 0.7$ (2 PCR probes, 0 NoPCR probe)<br>209604_s_at, 213134_x_at, 205548_s_at, 209603_at, 201826_s_at, 209602_s_at, 219051_x_at, 205339_at, 202392_s_at, 41660_at, 204862_s_at, 215867_x_at, 214164_x_at, 212046_x_at, 205425_at, 214058_at, 212207_at, 219209_at, 213033_s_at, 211302_s_at, 219664_s_at, 203139_at, 211939_x_at, 220016_at, 214383_x_at, 212721_at, 203453_at, 218065_s_at, 201755_at, 204825_at
$\alpha = 0.8$ (7 PCR probes, 0 NoPCR probe)<br>209604_s_at, 213134_x_at, 205548_s_at, 209603_at, 209602_s_at, 201826_s_at, 41660_at, 219209_at, 213033_s_at, 219051_x_at, 202392_s_at, 205339_at, 205425_at, 214058_at, 204862_s_at, 219664_s_at, 201649_at, 215867_x_at, 214164_x_at, 212046_x_at, 203453_at, 211939_x_at, 211302_s_at, 203227_s_at, 202088_at, 203139_at, 220044_x_at, 218164_at, 217867_x_at, 209551_at
$\alpha = 0.9$ (8 PCR probes, 0 NoPCR probe)<br>209604_s_at, 213134_x_at, 205548_s_at, 209602_s_at, 219209_at, 213033_s_at, 209603_at, 201826_s_at, 41660_at, 201649_at, 219051_x_at, 202392_s_at, 205425_at, 214058_at, 219664_s_at, 203453_at, 205339_at, 220044_x_at, 218164_at, 217867_x_at, 209551_at, 204124_at, 204862_s_at, 215867_x_at, 214164_x_at, 212046_x_at, 211939_x_at, 203227_s_at, 202088_at, 204750_s_at
$\alpha = 1$ (13 PCR probes, 0 NoPCR probe)<br>209604_s_at, 219209_at, 213033_s_at, 209602_s_at, 41660_at, 213134_x_at, 209603_at, 205548_s_at, 201826_s_at, 201649_at, 220044_x_at, 219664_s_at, 219051_x_at, 218164_at, 217867_x_at, 214058_at, 209551_at, 205425_at, 204124_at, 203453_at, 202392_s_at, 221957_at, 220702_at, 219984_s_at, 218352_at, 217833_at, 217566_s_at, 215867_x_at, 215078_at, 214164_x_at
