# Supplementary material for: Prediction of the outcome of preoperative chemotherapy in breast cancer using DNA probes that provide information on both complete and incomplete responses
Source: BMC Bioinformatics. 2008 Mar 15;9:149. doi: 10.1186/1471-2105-9-149 (PMC2292140; doi:10.1186/1471-2105-9-149)
Supplement: Additional file 8 — Table – Patients characteristics. The data provided give the patient characteristics for the 4 cohorts of the study. [file 1471-2105-9-149-S8.doc]

Supplemental Table 3 – Patients characteristics

|  | **training set** | **test set 1** | **test set 2** | **test set 3** |
| --- | --- | --- | --- | --- |
|  | MDACC | MDACC | Collaborative | IGR |
| **n** | 82 | 51 | 147 | 50 |
| **Age, years** |  |  |  |  |
| **Median** | 52 | 50 | 50 | 50 |
| **Range** | 29-79 | 28-73 | 26-75 | 31-75 |
| **TNM stage** |  |  |  |  |
| **T1** | 9% | 12% | 9% | 0% |
| **T2** | 56% | 47% | 58% | 58% |
| **T3** | 18% | 14% | 18% | 34% |
| **T4** | 17% | 27% | 15% | 0% |
| **Unknown** |  |  |  | 8% |
| **N0** | 34% | 23% | 24% | NA |
| **N1** | 46% | 49% | 46% | NA |
| **N2** | 10% | 12% | 15% | NA |
| **N3** | 10% | 16% | 15% | NA |
| **Nuclear grade** |  |  |  |  |
| **1** | 2% | 0% | 7% | 0% |
| **2** | 37% | 47% | 35% | 32% |
| **3** | 61% | 53% | 58% | 64% |
| **Unknown** |  |  |  | 4% |
| **ER positive^*^** | 43% | 69% | 48% | 36% |
| **ER negative** | 57% | 31% | 52% | 64% |
| **HER-2 positive** | 30% | 16% | 5% | 32% |
| | 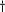**HER-2 negative** | | --- | | 70% | 82% | 95% | 68% |
| **Neoadjuvant therapy regimen** | TFAC | TFAC | TFAC | FEC |
| **Pathologic complete response** | 26% | 26% | 19% | 48% |
| **Residual disease** | 74% | 74% | 81% | 52% |
